# Supplementary material for: Novel fold of rotavirus glycan-binding domain predicted by AlphaFold2 and determined by X-ray crystallography
Source: Commun Biol. 2022 May 5;5:419. doi: 10.1038/s42003-022-03357-1 (PMC9072675; doi:10.1038/s42003-022-03357-1)
Supplement: Supplementary file 2 — Supplementary Material [file 42003_2022_3357_MOESM2_ESM.pdf]

Supplementary Information

Novel fold of rotavirus glycan-binding domain predicted by AlphaFold2 and determined by X-ray crystallography

Liya Hu[1*], Wilhelm Salmen[1], Banumathi Sankaran[2], Yi Lasanajak[3], David F. Smith[3], Sue E. Crawford[4], Mary K. Estes[4,5], B.V. Venkataram Prasad[1,2*]

[1]Verna and Marrs McLean Department of Biochemistry and Molecular Biology, Baylor College of Medicine, Houston, TX, USA

[2]Berkeley Center for Structural Biology, Molecular Biophysics and Integrated Bioimaging, Lawrence Berkeley Laboratory, Berkeley, CA, USA

[3]Emory Glycomics and Molecular Interactions Core (EGMIC), Emory University School of Medicine, Atlanta, GA, USA

[4]Department of Molecular Virology and Microbiology, Baylor College of Medicine, Houston, TX, USA

[5]Department of Medicine, Baylor College of Medicine, Houston, TX, USA

***Corresponding Authors:**

Liya Hu, Ph.D.: lhu@bcm.edu

B.V. Venkataram Prasad, Ph.D.: vprasad@bcm.edu

Supplementary Table 1 | Percent Identity Matrix created by Clustal 2.1[1].

**Group | Host | Strain | Accession**	**Percent Identity Matrix (%)**						
	RVB Human NIV-094456	RVB Human ADRV	RVB Murine IDIR	RVB Bovine DB176	RVB Porcine KS1	RVA Human DS1	RVC Human Bristol
RVB|Human|NIV-094456|AET79992	100	96.23	33.33	27.04	28.66	11.57	11.11
RVB|Human|ADRV|D6NGF7	96.23	100	34.59	27.67	28.66	11.57	11.11
RVB|Murine|IDIR|P15155	33.33	34.59	100	35.58	25	14.52	17.57
RVB|Bovine|DB176|ADC53102	27.04	27.67	35.58	100	32.1	11.9	14.67
RVB|Porcine|KS1|AUG45027	28.66	28.66	25	32.1	100	10.85	19.21
RVA|Human|DS1|AEG25325	11.57	11.57	14.52	11.9	10.85	100	18.29
RVC|Human|Bristol|Q82040	11.11	11.11	17.57	14.67	19.21	18.29	100

The virus group, host, strain, and database accession numbers are given.

Supplementary Table 2 | Top 10 glycans that bind to GST-tagged VP8*B.

ID	Glycan	Average RFU	Std	%CV
539	Galb1-4GlcNAcb1-3Galb1-4GlcNAcb1-3Galb1-4GlcNAcb1-2Mana1-6(Galb1-4GlcNAcb1-3Galb1-4GlcNAcb1-3Galb1-4GlcNAcb1-2Mana1-3)Manb1-4GlcNAcb1-4GlcNAcb-Sp12	2146	45	2
375	GalNAcb1-4GlcNAcb1-2Mana1-6(GalNAcb1-4GlcNAcb1-2Mana1-3)Manb1-4GlcNAcb1-4GlcNAc-Sp12	2077	217	10
550	Gala1-3Galb1-4GlcNAcb1-2Mana1-6(Gala1-3Galb1-4GlcNAcb1-2Mana1-3)Manb1-4GlcNAcb1-4GlcNAc-Sp24	1839	260	14
537	GlcNAcb1-3Galb1-4GlcNAcb1-3Galb1-4GlcNAcb1-2Mana1-6(GlcNAcb1-3Galb1-4GlcNAcb1-3Galb1-4GlcNAcb1-2Mana1-3)Manb1-4GlcNAcb1-4GlcNAcb-Sp12	1531	93	6
596	Neu5Aca2-6Galb1-4GlcNAcb1-3Galb1-4GlcNAcb1-3Galb1-4GlcNAcb1-2Mana1-6(Neu5Aca2-6Galb1-4GlcNAcb1-3Galb1-4GlcNAcb1-3Galb1-4GlcNAcb1-2Mana1-3)Manb1-4GlcNAcb1-4GlcNAcb-Sp12	1179	62	5
581	Galb1-4GlcNAcb1-3Galb1-4GlcNAcb1-6(Galb1-4GlcNAcb1-3Galb1-4GlcNAcb1-3)GalNAca-Sp14	932	46	5
565	Galb1-4GlcNAcb1-3Galb1-4GlcNAcb1-2Mana1-6(Galb1-4GlcNAcb1-3Galb1-4GlcNAcb1-2Mana1-3)Manb1-4GlcNAcb1-4(Fuca1-6)GlcNAcb-Sp24	903	77	9
207	Mana1-2Mana1-2Mana1-3Mana-Sp9	781	235	30
597	Neu5Aca2-3Galb1-4GlcNAcb1-3Galb1-4GlcNAcb1-3Galb1-4GlcNAcb1-2Mana1-6(Neu5Aca2-3Galb1-4GlcNAcb1-3Galb1-4GlcNAcb1-3Galb1-4GlcNAcb1-2Mana1-3)Manb1-4GlcNAcb1-4GlcNAcb-Sp12	742	28	4
548	GlcNAcb1-3Galb1-4GlcNAcb1-6(GlcNAcb1-3Galb1-4GlcNAcb1-2)Mana1-6(GlcNAcb1-3Galb1-4GlcNAcb1-2Man a1-3)Manb1-4GlcNAcb1-4GlcNAc-Sp24	710	45	6

Glycan Array results for GST-VP8*B. The strength of binding of the protein to the glycan is represented using the relative fluorescence units (RFU) of binding. Standard deviation (Std) and % Coefficient of variation (%CV) from the mean of 6 replicates of each glycan printed on the array is provided. The glycans are arranged in descending order of RFU.

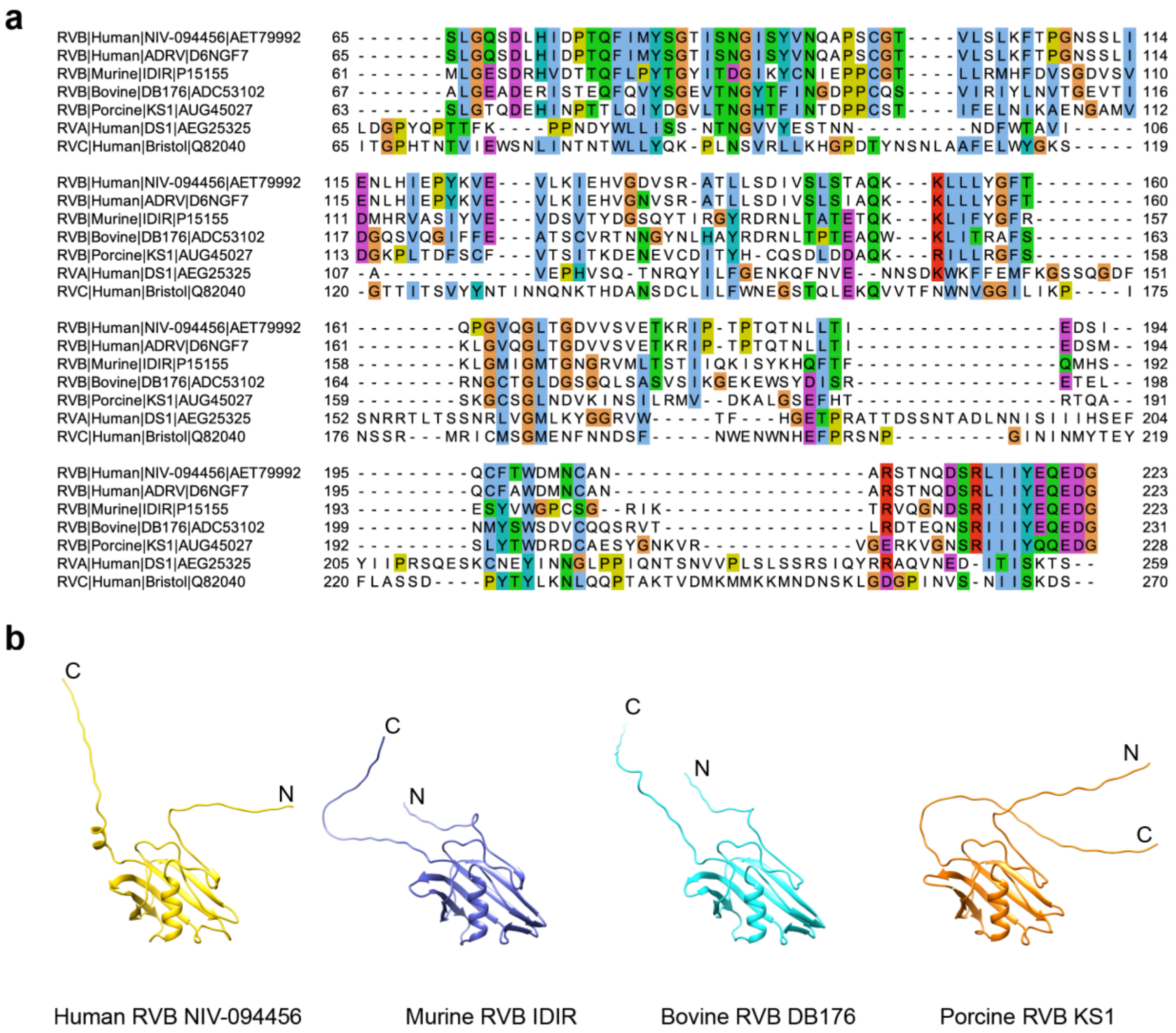

Supplementary Figure 1 | Sequence alignment of groups A, B, and C VP8*s and comparison of VP8*B AlphaFold2 models. a, Sequence alignment of VP8*s of human and animal RVBs and the human RVA and RVC strains. The amino acids are colored using the Clustal protein color scheme in Jalview[2]. **b**, The AlphaFold2 models of VP8*B of human, murine, bovine, and porcine group B rotaviruses. The models are superimposed and shown side by side for clarity.

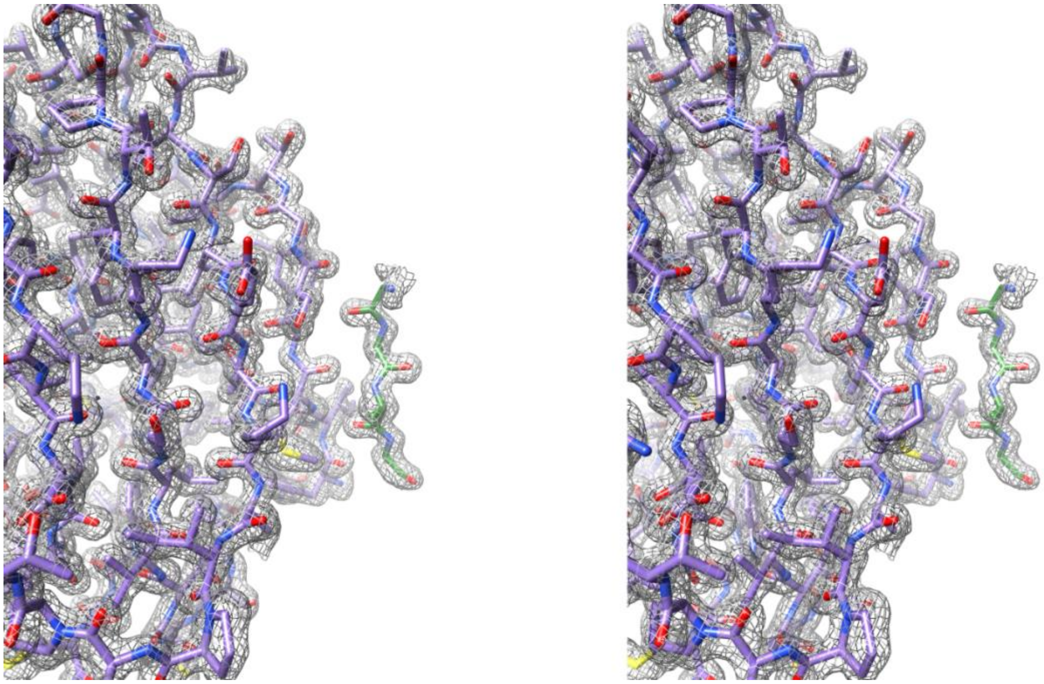

Supplementary Figure 2 | Cross-eye stereo image of the electron density map of VP8*B. The portion that contains part of chain B (purple) and the short peptide (green) are shown as stick models with heteroatoms colored by elements: oxygen, red; nitrogen, blue. The experimental 2mFo-DFc electron map is shown as gray mesh contoured at 1 σ.

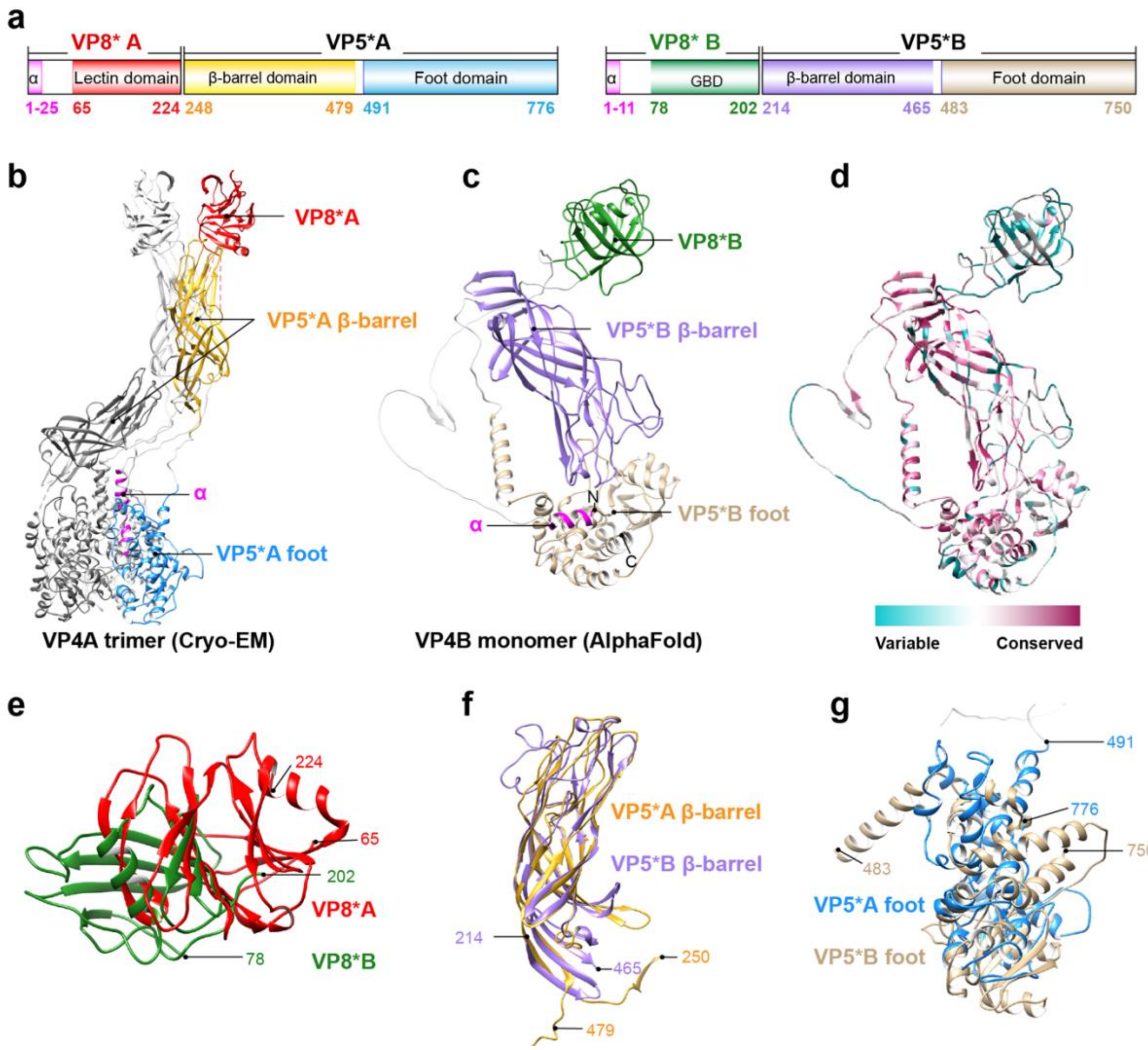

Supplementary Figure 3 | ab initio modeling full-length VP4B with AlphaFold2. a, The schematic of VP4A and VP4B domains. **b,** Cryo-EM structure of RVA VP4 trimers (PDB ID: 4V7Q). One of the RVA VP4 chains is colored (N-terminal α-helix, magenta; VP8*, red; VP5* β-barrel domain, gold; VP5* foot domain, blue). The other two VP4 chains are shown in white and dark grey, respectively. **c**, AlphaFold2 model of RVB VP4 is shown in the ribbon diagram. The VP8*B, β-barrel domain of VP5*B, and the foot domain of VP5*B are colored in green, purple, and tan, respectively. **d,** ConSurf analysis of VP4B[3]. Residue conservation is plotted onto the ribbon diagram of VP4B AlphaFold model and is colored according to conservation scores

from low conservation (cyan) to conserved (dark purple). **e-g,** Overlay of VP8*, VP5 β-barrel and foot domains of RVB and RVA. Each domain is colored as in a-c and labeled.

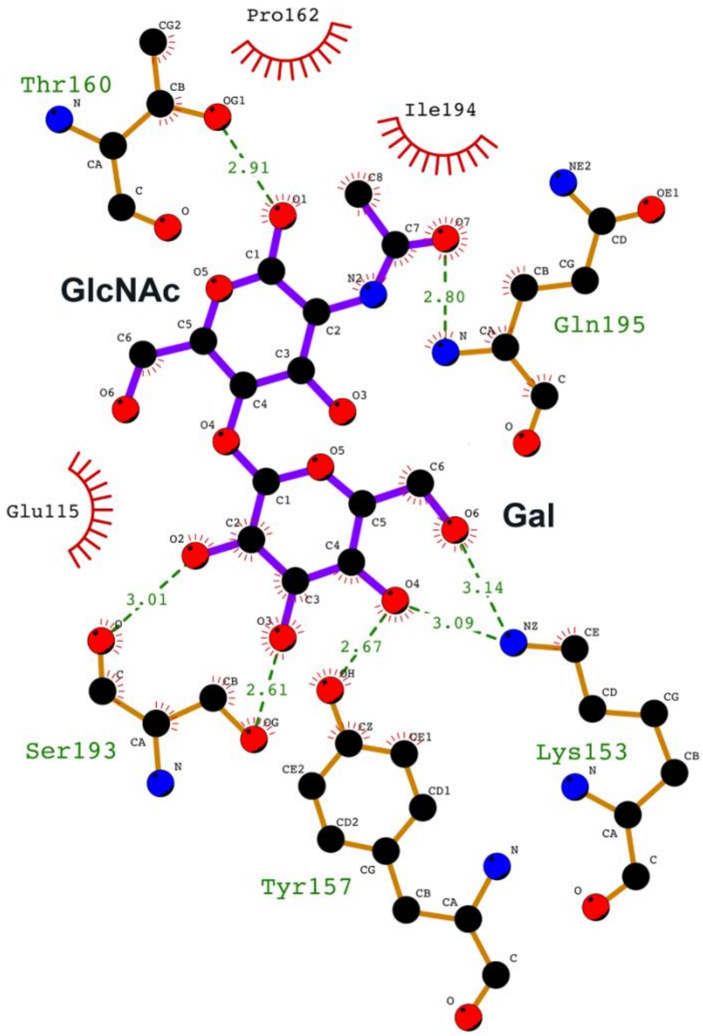

Supplementary Figure 4 | Ligplot analysis of the interactions between VP8*B and LacNAc[4]. The amino acid and the glycan residues involved in the interactions are labeled. The carbon, nitrogen, and oxygen atoms are shown in black, blue, and red-colored circles, respectively. Hydrogen bond interactions are represented as green dashed lines between the respective donor and acceptor atoms along with the bond distance. An arc indicates the van der Walls contacts with radiating spokes.

Supplementary References

1 Madeira, F. *et al.* The EMBL-EBI search and sequence analysis tools APIs in 2019. *Nucleic Acids Res* **47**, W636-W641, doi:10.1093/nar/gkz268 (2019).

2 Waterhouse, A. M., Procter, J. B., Martin, D. M., Clamp, M. & Barton, G. J. Jalview Version 2--a multiple sequence alignment editor and analysis workbench. *Bioinformatics* **25**, 1189-1191, doi:10.1093/bioinformatics/btp033 (2009).

3 Ashkenazy, H. *et al.* ConSurf 2016: an improved methodology to estimate and visualize evolutionary conservation in macromolecules. *Nucleic Acids Res* **44**, W344-350, doi:10.1093/nar/gkw408 (2016).

4 Laskowski, R. A. & Swindells, M. B. LigPlot+: multiple ligand-protein interaction diagrams for drug discovery. *J Chem Inf Model* **51**, 2778-2786, doi:10.1021/ci200227u (2011).
