# Supplementary material for: Novel fold of rotavirus glycan-binding domain predicted by AlphaFold2 and determined by X-ray crystallography
Source: Commun Biol. 2022 May 5;5:419. doi: 10.1038/s42003-022-03357-1 (PMC9072675; doi:10.1038/s42003-022-03357-1)
Supplement: Supplementary file 3 — Description of Additional Supplementary Files [file 42003_2022_3357_MOESM3_ESM.pdf]

Description of Additional Supplementary Files

File name: Supplementary Data 1
Description: Glycan array data for VP8*B.
